# Supplementary material for: Rapid Prototyping of Multi-Functional and Biocompatible Parafilm®-Based Microfluidic Devices by Laser Ablation and Thermal Bonding
Source: Micromachines (Basel). 2023 Mar 14;14(3):656. doi: 10.3390/mi14030656 (PMC10054776; doi:10.3390/mi14030656)
Supplement: Supplementary file 1 [file micromachines-14-00656-s001.zip › micromachines-2208782-supplementary.pdf]

# Supplementary Material

## Rapid prototyping of multi-functional and biocompatible Parafilm®-based microfluidic devices by laser ablation and thermal bonding

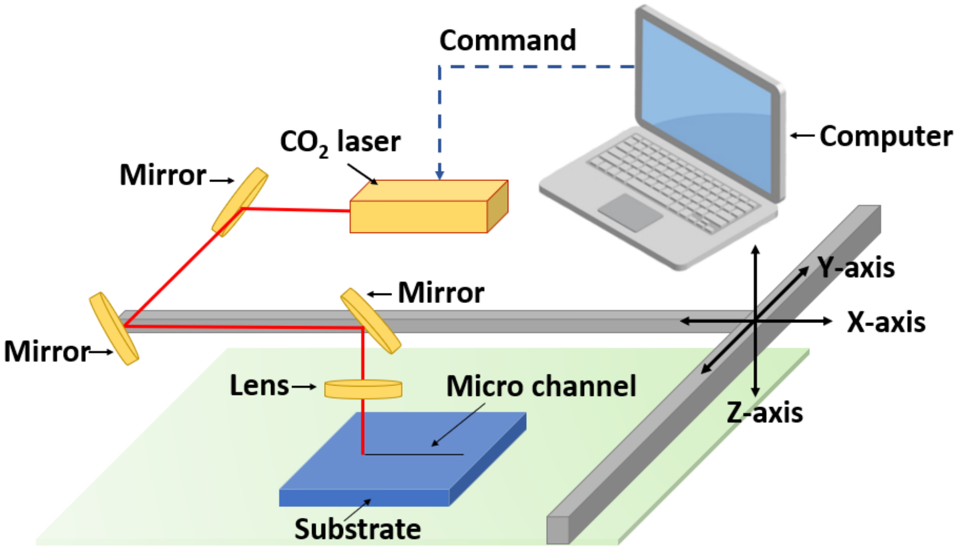


**Supplementary Figure S1** Laser ablation setup for patterning Parafilm® and different microfluidic substrates.

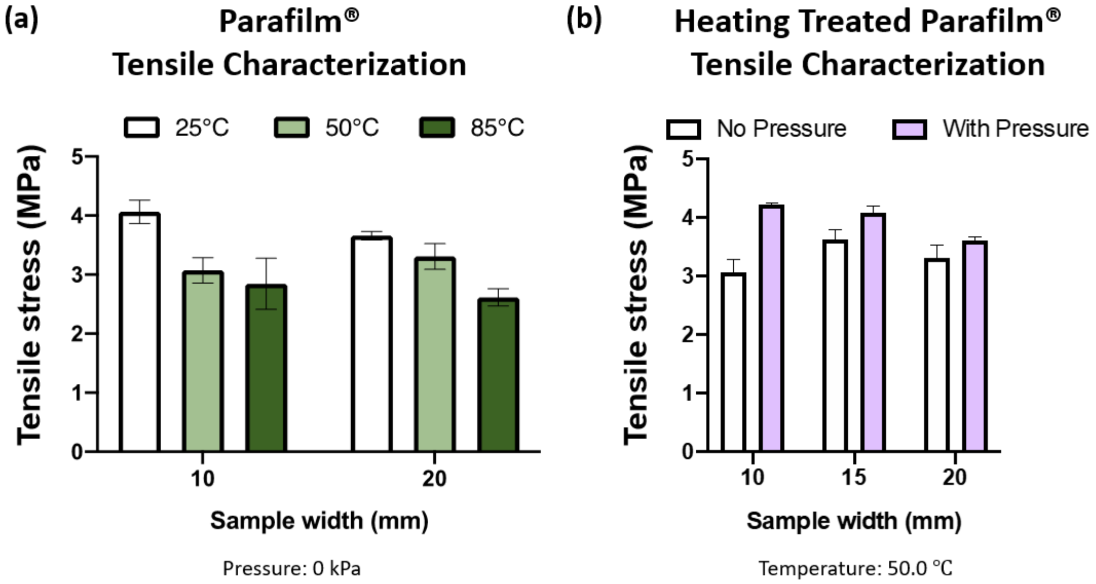


**Supplementary Figure S2** Effects of temperature and pressure on Parafilm® mechanical properties and deformation. (a) Tensile stress results of Parafilm® with different gauge widths (10 mm and 20 mm) with the same trend as the 15 mm Parafilm® specimen. (b) Parafilm® applied 0.4 kPa static pressure has higher ultimate tensile stress, indicating better elasticity. The above results show the capability of Parafilm® to perform as a functional membrane.

Please do not adjust margins

Please do not adjust margins



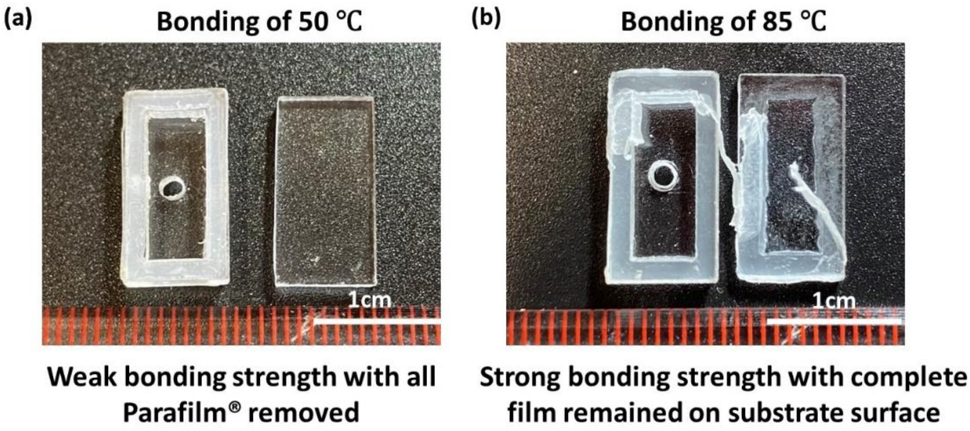


**Supplementary Figure S3** Images of PbLT fabricated microfluidics chips after the gas-burst test. Scale bar: 1cm. The cross-section of chips fabricated with a thermal bonding temperature of 50℃ (a) is clean, showing weak bonding strength. When the injected gas pressure arrives at 0.1MPa, gas leaking was observed and all Parafilm® was removed. The cross-section of 85℃ (b) has obvious residue and adhesion indicating high bonding strength and good sealing ability.

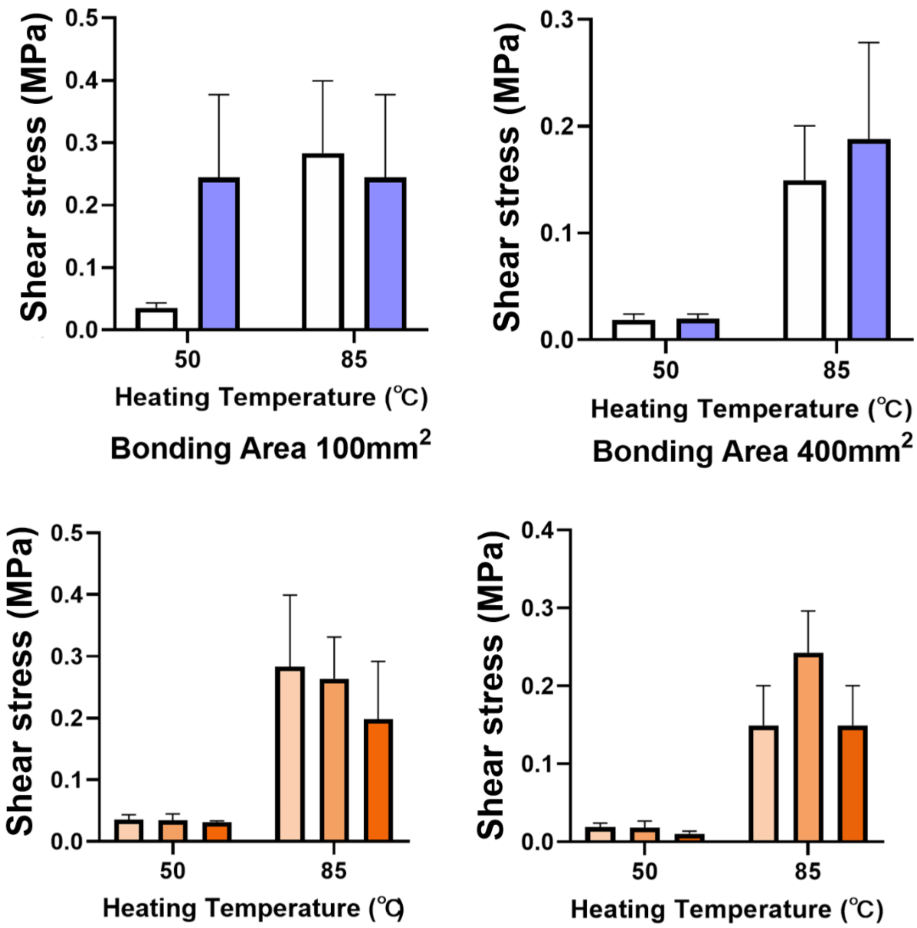


**Supplementary Figure S4** Shear stress for PbLT biochips with thermal bonding temperatures of 50.0 °C and 85.0 °C. (a) After incubation, an evident decrease in the bonding strength (45.9%) was observed for the biochips fabricated at 85°C. For A biochips fabricated at 50.0 °C, the change was a slight increase presumably due to the effects of the humidity environment. (b) The bonding strength of PVC substrates and glass substrates was comparable to that of PMMA. Chips fabricated at 85.0 °C have larger bonding strength than the ones at 50.0 °C.



This journal is © The Royal Society of Chemistry 20xx

Please do not adjust margins

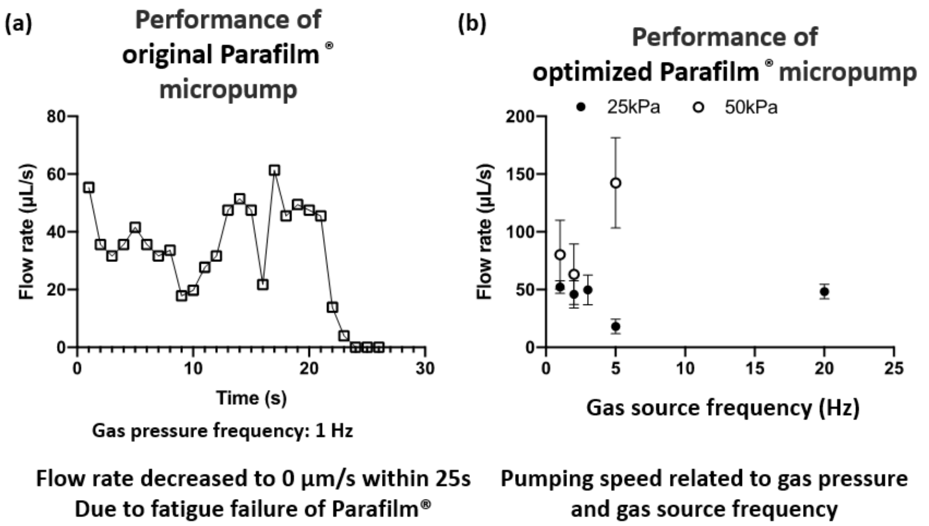


**Supplementary Figure S5** Characterization of PbLT fabricated micropump. (a) For the type 1 chip (utilizing original Parafilm® as the functional membrane), the flow rate decreased from over 20.0 μL/s to 0 μL/s within 25s, which presumably dues to the fatigue failure of Parafilm®. (b)Pumping Performance of optimized Parafilm® micropump related to actuation gas pressure and gas source frequency.

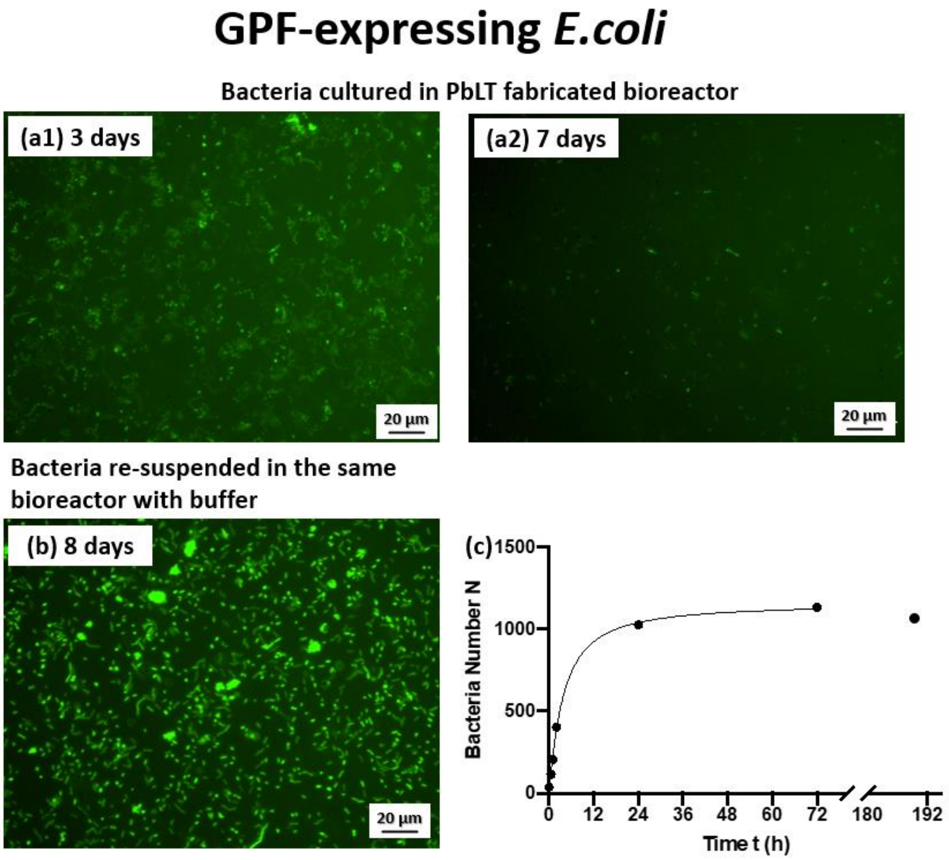


**Supplementary Figure S6** Fluorescence images acquired at 3-day (a1), 7-day(a2), post-cultivation in the bioreactor, 8-day after bacteria re-suspended in the same bioreactor (b) and plotted growth curve (c) (Scale bar = 20 μm). When nutrients were consumed at 3-day and 7-day post-cultivation in a confined space, the green fluorescence was significantly decreased owing to the death of E. coli. After adding nutrients at 8-day, the bacteria re-proliferated.

This journal is © The Royal Society of Chemistry 20xx


Please do not adjust margins

Please do not adjust margins



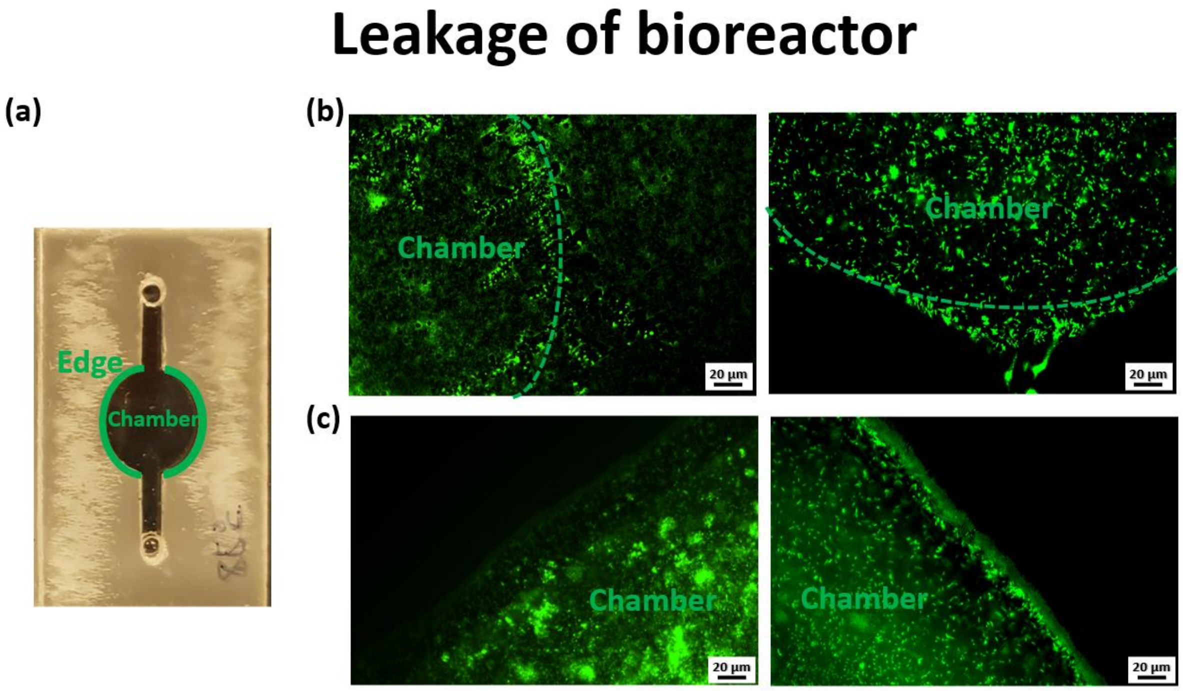


**Supplementary Figure S7** Leakage photos of PbLT-fabricated bioreactor. (a) A top view of the fabricated bioreactor. (b) 3 out of 15 PbLT fabricated bioreactors leaked soon after injecting the medium. (c) No leakage has been observed for the rest bioreactors after 7-day cultivation. (Scale bar = 20µm)

**Supplementary Table S1**.

The thickness of 1) received Parafilm®, 2) Stretched Parafilm®, and 3) Parafilm® after bonding.

<table>
<tr><td colspan="2" rowspan="2"></td><td colspan="4">The thickness of Parafilm® (µm)</td></tr>
<tr><td>1</td><td>2</td><td>3</td><td>Mean</td></tr>
<tr><td colspan="2">Received (original) Parafilm®</td><td>145.59</td><td>142.32</td><td>144.06</td><td><u>143.99</u></td></tr>
<tr><td colspan="2">Stretched Parafilm®</td><td>42.69</td><td>47.76</td><td>51.13</td><td><u>47.19</u></td></tr>
<tr><td colspan="6">Parafilm® after bonding</td></tr>
<tr><td colspan="2">Bonding condition</td><td rowspan="2">1</td><td rowspan="2">2</td><td rowspan="2">3</td><td rowspan="2">Mean</td></tr>
<tr><td>Temperature（°C）</td><td>Pressure (kPa)</td></tr>
<tr><td rowspan="3">85</td><td>8.3</td><td>100.48</td><td>119.35</td><td>115.59</td><td><u>111.81</u></td></tr>
<tr><td>16.7</td><td>101.12</td><td>107.40</td><td>105.30</td><td><u>104.61</u></td></tr>
<tr><td>33.3</td><td>100.81</td><td>105.04</td><td>103.79</td><td><u>103.21</u></td></tr>
</table>



This journal is © The Royal Society of Chemistry 20xx

Please do not adjust margins

**Supplementary Table S2**.

Comparison of PbLT and conventional bonding methods. PbLT provides higher bonding strength and supports more substrates.

| Method | Bonding process | Bonding strength (MPa) | Supported substrates | Ref |
|---|---|---|---|---|
| **PbLT (this work)** | **Thermal fusion bonding at 85°C** | **0.2 (Gas burst pressure)** | **Thermal Plastics (PMMA, PVC), glass** | **Our work** |
| | **Thermal fusion bonding at 50°C** | **0.06 (Gas burst pressure)** | **Thermal Plastics (PMMA, PVC), glass** | |
| PDMS-PDMS bonding | Plasma-assisted bonding | 0.2 | PDMS | [1] |
| | Oxygen Plasma | 0.3 | PDMS | [2] |
| | Corona treatment | 0.3 | PDMS | |
| | UV illumination | 0.1 | PDMS | [3] |
| PDMS bonding to various substrates | Plasma treatment and surface modification | 3.0 | PMMA | [4] |
| | Corona discharge | 0.23-0.38 | Glass | [4] |
| | PDMS pre-polymer gluing | > 0.5 | Glass | [3] |


1. Lu, Y.; Shi, Z.; Yu, L.; Li, C.M. Fast Prototyping of a Customized Microfluidic Device in a Non-Clean-Room Setting by Cutting and Laminating Parafilm®. *RSC Adv.* **2016**, *6*, 85468–85472.
2. Eddings M A, Johnson M A, Gale B K. Determining the optimal PDMS–PDMS bonding technique for microfluidic devices. *Journal of Micromechanics and Microengineering*, **2008**, 18(6): 067001.
3. Yousuff, C.M.; Danish, M.; Ho, E.T.W.; Basha, I.H.K.; Hamid, N.H.B. Study on the Optimum Cutting Parameters of an Aluminum Mold for Effective Bonding Strength of a PDMS Microfluidic Device. *Micromachines* **2017**, *8*.
4. Borók, A.; Laboda, K.; Bonyár, A. PDMS Bonding Technologies for Microfluidic Applications: A Review. *Biosensors* **2021**, *11*.




Please do not adjust margins
